# Erlotinib protests against LPS-induced parthanatos through inhibiting macrophage surface TLR4 expression

**DOI:** 10.1038/s41420-021-00571-4

**Published:** 2021-07-16

**Authors:** Qiong Xue, Xiaolei Liu, Cuiping Chen, Xuedi Zhang, Pengyun Xie, Yupin Liu, Shuangnan Zhou, Jing Tang

**Affiliations:** 1 The Department of Anesthesiology, Pain and Perioperative Medicine, The First Affiliated Hospital of Zhengzhou University, Zhengzhou, Henan China; 2 The Department of Anesthesiology, Affiliated Hospital of Guangdong Medical University, Zhanjiang, Guangdong China; 3 Department of Medical Iconography, The Second Affiliated Hospital University of Guangzhou Traditional Chinese Medicine, Guangzhou, Guangdong China; 4 Department of Liver Diseases, Fifth Medical Center of Chinese PLA General Hospital, Beijing, China

**Keywords:** Molecular biology, Apoptosis

## Abstract

Sepsis is a life-threatening cascading systemic inflammatory response syndrome on account of serve infection. In inflamed tissues, activated macrophages generate large amounts of inflammatory cytokines reactive species, and are exposed to the damaging effects of reactive species. However, comparing with necroptosis and pyroptosis, so far, there are few studies focusing on the overproduction-related cell death, such as parthanatos in macrophage during sepsis. In LPS-treated macrophage, we observed PARP-1 activation, PAR formation and AIF translocation. All these phenomena could be inhibited by both erlotinib and 3-AB, indicating the presence of parthanatos in endotoxemia. We further found that LPS induced the increase of cell surface TLR4 expression responsible for the production of ROS and subsequent parthanatos in endotoxemia. All these results shed a new light on how TLR4 regulating the activation of PARP-1 by LPS in macrophage.

## Introduction

In the circumstance of DNA damage, nuclear enzyme, poly (ADP-ribose) (PAR) polymerase-1 (PARP-1), activates and facilitates DNA repair [1]. Excessive activation of PARP-1 depletes cellular NAD+ and slows down ATP formation, resulting in the formation of PAR polymers. As a result, apoptosis-inducing factor (AIF) translocates from mitochondria into cytoplasm, launching a caspase-independent cell death program, called parthanatos (PARP-1-dependent cell death) [2–4]. Some studies demonstrated that reactive oxygen species (ROS) is critical to the process of parthanatos after genotoxic agents and treatment [5–7]. Oxidative stress/ROS overproduction is an important initiator. So far, parthanatos has been reported to participate in many diseases, such as ischemia-reperfusion injury, neurobiological disorders, and inflammatory injury [8–11].

Toll-like receptor (TLR) family belongs to type I transmembrane pattern recognition receptors and plays a fundamental character in sensing invading causative agents or endogenous damage signals. Of them, toll-like receptor 4 (TLR4) is widely studied and the key receptor for recognizing bacterial component called lipopolysaccharide (LPS) [12, 13]. An amplified ROS production from LPS-primed is the result of MyD88-dependent signaling pathways following TLR4 activation [14, 15]. Recent studies have revealed that TLR4 alone is not sufficient for an overall innate immune response and requires coordination through CD14 and MD2 [16, 17]. Our research group also found that the improvement of TLR4 function requires the participation of epidermal growth factor receptor (EGFR) [18].

As a small molecule tyrosine kinase inhibitor of EGFR, erlotinib is widely used as anticarcinogen, particularly for some type of advanced pancreatic cancer and non-small cell lung cancer [19–21]. Also, several studies have shown that it is erlotinib that protects mice from LPS-induced endotoxicity [22, 23]. Similarly, our previous study found that erlotinib protests against inflammatory injury by inhibiting LPS-induced TLR4 phosphorylation [18]. However, the effect of ROS production induced by LPS processing on PARP-1 activation, the occurrence of parthanatos related to TLR4, and the mechanisms of EGFR involved remain unclear.

Here, we further explore the character of EGFR on the pathophysiological process of the development of parthanatos in endotoxemia. We found that not only MNNG, a classical DNA-alkylating agent, but also LPS could induce PARP-1 dependent parthanatos, and these effects could be inhibited by 3-AB, a PARP-specific inhibitor. Besides 3-AB, erlotinib also protects macrophage from parthanatos at least through two aspects. First, erlotinib decreases the phosphorylation of ERK1/2 through PI3K/AKT signal pathway since 3 h after LPS treatment. Then, erlotinib downregulated the expression of TLR4 on cell surface both in vitro and in vivo since 24 h after LPS treatments, which is critical for LPS-induced parthanatos.

## Results

### Erlotinib and 3-AB increase survival of mice or reduce cell death rate treated with LPS

**Fig. 1:**
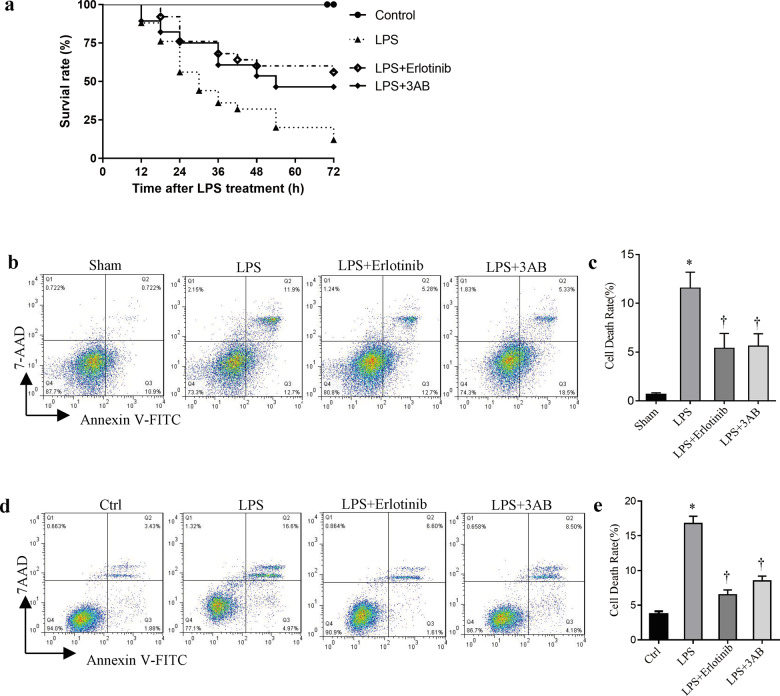
Effects of erlotinib and 3-AB on mice or cells treated with LPS. Wild-type C57BL/6 mice were primed with 100 mg kg^–1^ of erlotinib gavage administration or 3-AB before LPS treatment as described in methods. **a** After LPS stimulation, survival rate of mice was detected every 6 h for 72 h. LPS + erlotinib group (*n* = 20) and LPS + 3-AB group (*n* = 20)’s life span was significantly increased compared to LPS group (*n* = 20, *P* < 0.05). **b**, **c** After 24 h LPS treatment, peritoneal macrophages were collected and identified with F4/80. Cell death was analyzed by flow cytometry. **d**, **e** RAW264.7 cells were treated with LPS for 24 h in the presence or absence of pretreatment of erlotinib or 3-AB for 30 min followed by flow cytometry analysis of cell death. Error bars represent SD (*n* = 3). *P < 0.05 as compared with Sham; ^†^*P* < 0.05 as compared with the time-matched LPS group.

Although some studies have demonstrated that the main ways of cell death induced by LPS are necrosis and pyroptosis. So far, overproduction-related cell death, parthanatos, has not been reported in macrophage during sepsis. Here, the survival rates of control, LPS alone (20 mg/kg i.p.), and LPS plus erlotinib (100 mg/kg B.W.) and LPS plus 3-AB (30 mg/kg i.p.) group were studied. As shown in Fig. 1a, 72 h after treatment, only 16% of mice in LPS group were survived. On the contrary, LPS-injected mice pretreated with erlotinib had a higher survival rate of 52%. In addition, 3-AB pretreatment also significantly improved survival in LPS-treated mice with indicating that PARP-1 might play a critical role in endotoxemia. To further confirm these results in vivo, peritoneal lavage fluid macrophages were collected from normal and endotoxemic mice with or without erlotinib and 3-AB pretreatment through surface marker F4/80. As shown in Fig. 1b, c, compared with control group, the death rate of peritoneal macrophages from endotoxemic mice increased from 0.722 to 11.9%. Both erlotinib and 3-AB pretreatment could significantly alleviate cell death in peritoneal macrophages of endotoxemic mice. We also got similar results in a mouse macrophage cell line RAW264.7 cells in vitro (Fig. 1d, e).

### Erlotinib attenuates LPS-induced parthanatos in vivo and in vitro

**Fig. 2:**
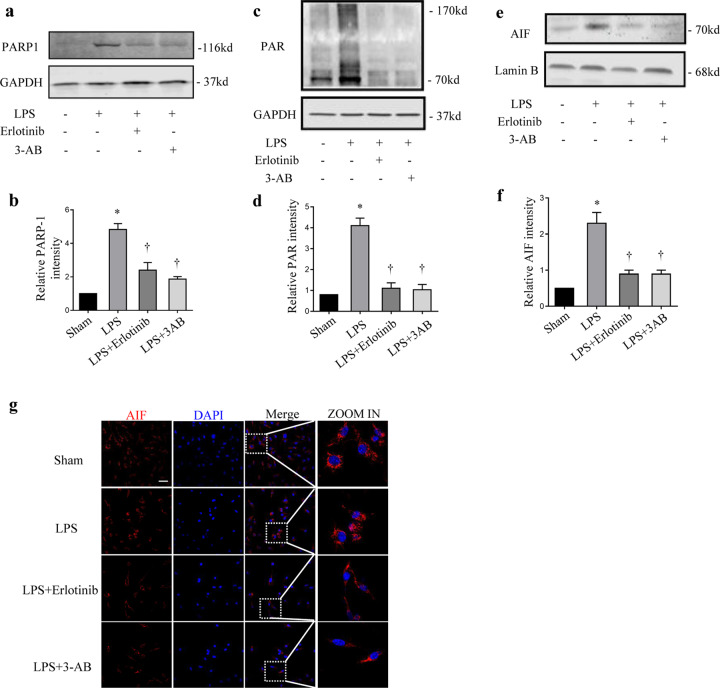
Effect of erlotinib on LPS-induced parthanatos. Macrophages collected from peritoneal lavage at 24 h after pretreated with erlotinib or 3-AB with LPS treatment. **a**, **c** Immunoblotted for PARP-1 activation, PAR formation, and GAPDH. **b**, **d** Mensuration of PARP-1 (**b**) and PAR (**d**) expressions. **e** Immunoblot analysis of AIF and Lamin B amount in nuclei. **f** Mensuration of AIF expression in nuclei. **g** Fluorescence images depicting AIF translocation (up panel; scale bar, 100 μm). Error bars represent SD (*n* = 3). *P < 0.05 as compared with NC; ^†^*P* < 0.05 as compared with LPS group.

**Fig. 3:**
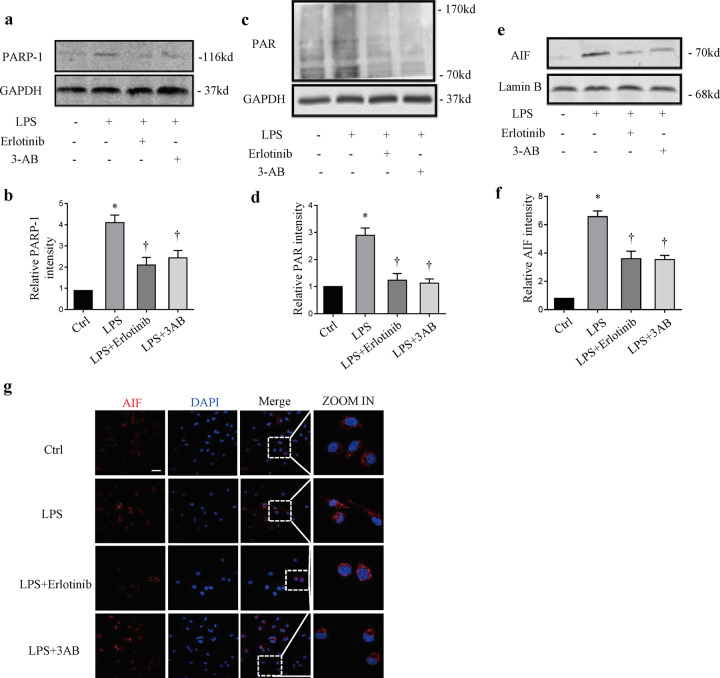
Erlotinib could inhibit LPS-induced parthanatos in RAW264.7 cells. RAW264.7 cells were pretreated with erlotinib (20 μM) or 3-AB (50 μM) with LPS (1 μg/ml) treatment. **a**, **c** Immunoblotted for PARP-1 activation, PAR formation and GAPDH. **b**, **d** Mensuration of PARP-1 (**b**) and PAR (**d**) expressions. **e** Immunoblot analysis of AIF and Lamin B amount in nuclei. **f** Mensuration of AIF expression. **g** Fluorescence images indicated that erlotinib and 3-AB pretreatment limited the AIF translocation (up panel; scale bar, 100 μm). Error bars represent SD (*n* = 3). *P < 0.05 as compared with NC; ^†^*P* < 0.05 as compared with LPS group.

To further the character of EGFR on parthanatos, wild-type (WT) C57BL/6 mice were treated with LPS with or without erlotinib or 3-AB pretreatment. As we expected LPS-induced PARP-1 activation, PAR formation and translocation of AIF into the nucleus could be inhibited by erlotinib indicating that EGFR may play a role in LPS-induced parthanatos (Fig. 2a–g). To confirm the ability of erlotinib in inhibiting parthanatos process, we used an in vitro system. RAW264.7 cells were pretreated with erlotinib or 3-AB 30 min before LPS treatment. Just as we expected, LPS-induced PARP-1 activation, PAR formation, and translocation of AIF into the nucleus could be inhibited by erlotinib (Fig. 3a–g). All these data suggest that erlotinib inhibits parthanatos both in vivo and in vitro.

### Erlotinib downregulates the surface expression of TLR4

**Fig. 4:**
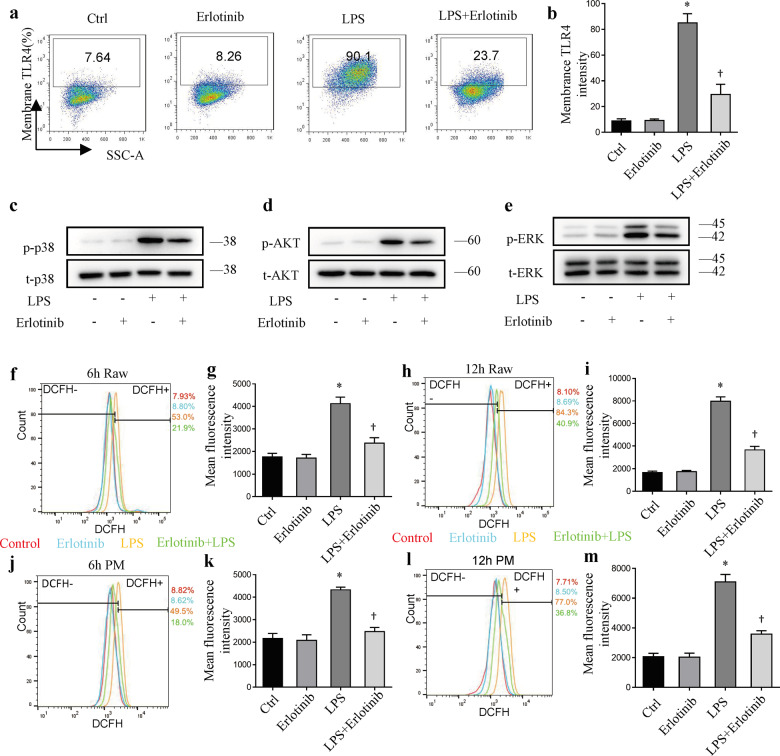
Cell surface TLR4 expression in response to LPS could be inhibited by erlotinib. **a**, **b** Cell surface TLR4 expression in RAW264.7 cells was analyzed by flow cytometry. **c**–**e** Immunoblot analysis of p-p38, t-p38, p-ERK1/2, t-ERK1/2, p-AKT, t-AKT in RAW264.7 cells treated with LPS for 3 h with or without erlotinib pretreatment for 30 min. **f**, **h**, **j**, **l** ROS production was measured with DCFH2-DA by FACS analysis. **g**, **i**, **k**, **m** Mean fluorescence intensity of ROS production. Error bars represent SD (*n* = 3). *P < 0.05 as compared with NC; ^†^*P* < 0.05 as compared with LPS group.

TLR4/MyD88 or TLR4/PI3K signaling pathway plays a vital role in the regulation of inflammatory. Our previous study reported that EGFR activation increases the cell surface TLR4 expression, indicating EGFR may also play a role in parthanatos in response to LPS [18]. Pretreatment of RAW264.7 cells with erlotinib for 30 min prior to LPS effectively inhibited the expression of TLR4 on cell surface at 24 h after LPS treatment (Fig. 4a, b). Immunoblot assay was performed to detect the level of p38, p-p38 (Fig. 4c), AKT, p-AKT (Fig. 4d), ERK1/2, and p-ERK1/2 (Fig. 4e). Compared with the negative control group, the upregulated expressions of p-p38, p-AKT, and p-ERK1/2 were response to LPS. However, erlotinib treatment reversed the changes of these proteins in LPS-induced RAW264.7 cells. Next, we measured the variation of ROS with fluorescent probe 29-,79-dichlorofluorescein diacetate (DCFH2-DA) after LPS treatment. As shown in Fig. 4f–i, compared with LPS group, erlotinib pretreatment could partially inhibit the production of ROS at 6 and 12 h. In peritoneal macrophage, erlotinib also reduced LPS-induced ROS production (Fig. 4j–m). These results elucidated the mechanism of LPS-induced inflammatory response, which was shown to be prevented by inhibiting surface TLR4 translocation.

### TLR4 receptor is required for LPS-induced parthanatos

**Fig. 5:**
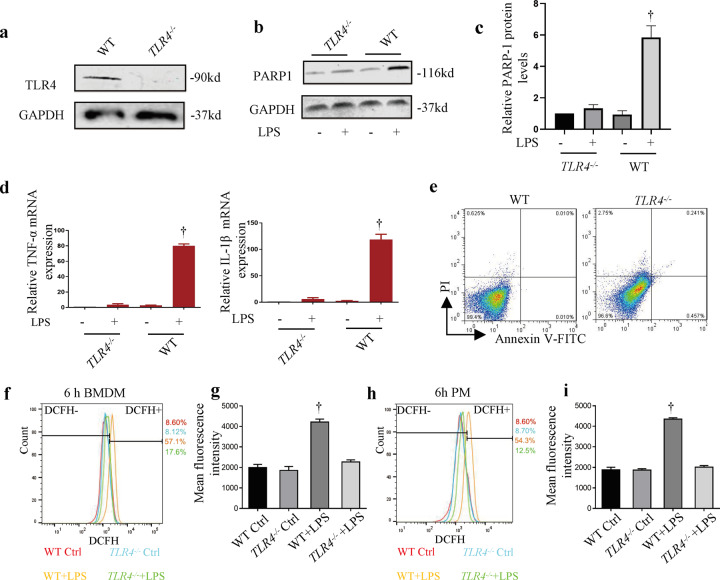
Downregulated cell surface TLR4 expression inhibits LPS-induced PARP-1 activation. BMDM cells were untreated or primed with LPS (1 μg ml^–1^) for 12 h. **a** Western blot analysis of TLR4 from WT and *TLR4*^*–/–*^ mice BMDM cells. **b** PARP-1 and GAPDH protein levels. **c** Mensuration of PARP-1 expressions. **d** WT and *TLR4*^*–/–*^ BMDM cells were treated with LPS for 6 h following by real-time PCR analysis of TNF-α and IL-1β mRNA expression. **e** BMDM cells were treated with LPS for 24 h followed by flow cytometry analysis of cell death. **f**, **h** ROS production was measured with DCFH2-DA by FACS analysis. **g**, **i** Mean fluorescence intensity of ROS production. Error bars represent SD (*n* = 3). ^†^*P* < 0.05 as compared with *TLR4*^*–/–*^ + LPS group.

The involvement of TLR4 in LPS-induced PARP-1 activation promoted us to focus on understanding whether TLR4 translocation might trigger parthanatos. To study how LPS actives parthanatos in macrophage, TLR4 knockout mice were used to verify the hypothesis. Figure 5a shows the results of genetic identification of TLR4 knockout mice. Accordingly, we show that bone marrow-derived macrophage (BMDM) from *TLR4*^*–/–*^ mice treated with LPS for 12 h failed to stimulate PAPR-1 activation compared with WT BMDM cells (Fig. 5b, c). Meanwhile, LPS significantly increased WT BMDM TNF-α and IL-1β mRNA expression at 6 h, and this was unobserved in *TLR4*^*–/–*^ BMDM (Fig. 5d). Also, knockout TLR4 did not affect cell death (Fig. 5e). In addition, we investigated the ROS production in WT or *TLR4*^*–/–*^ BMDM (Fig. 5f, g) and peritoneal macrophage (Fig. 5h, i). LPS-induced ROS production was dramatically decreased in *TLR4*^*–/–*^ BMDM. Similarly, compared with WT mice, the expression of ROS in the peritoneal macrophages of *TLR4*^*–/–*^ mice was effectively decreased. To sum up, these results suggest that TLR4 is involved in LPS-induced parthanatos.

## Discussion

**Fig. 6:**
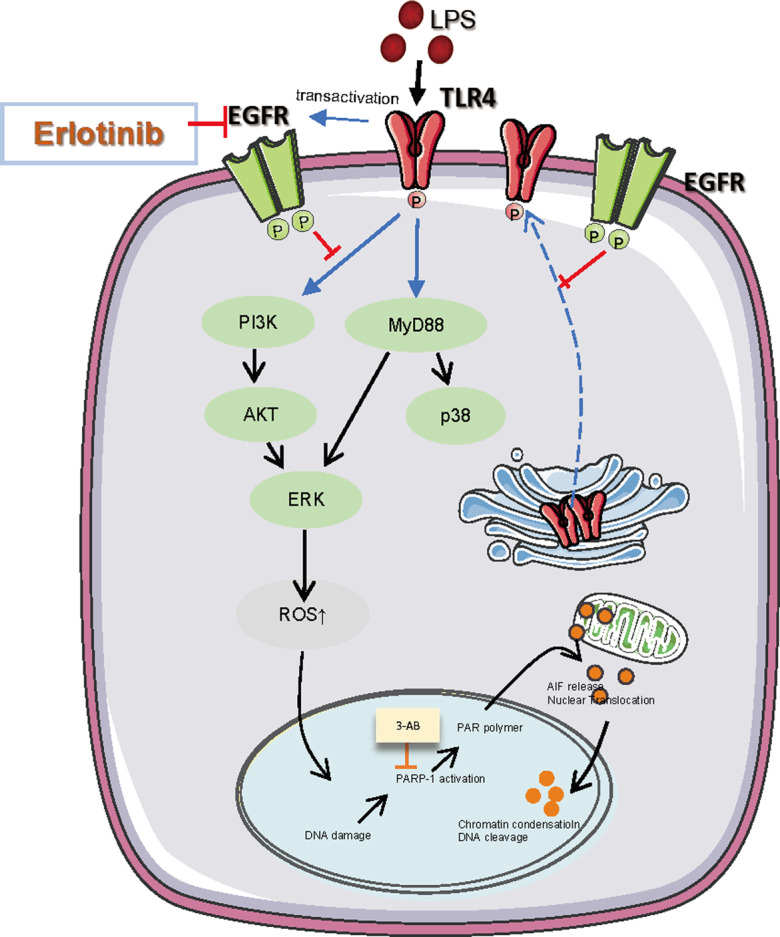
The role of TLR4 in parthanatos and the MyD88 and/or the PI3K signaling pathway in parthanatos. One of the reasons that LPS-induced ROS production leads to parthanatos. Erlotinib could inhibit the cells surface TLR4 expression and alleviated the parthanatos in response to LPS. This may be through the MyD88 and/or the PI3K signaling pathway. Besides, erlotinib inhibits the generation of ROS, which in turns inhibits parthanatos.

Sepsis or endotoxemia is a potential condition characterized by a cascade of events leading to multiorgan dysfunction to failure and ultimately death. According to related publication, parthanatos has not been reported in sepsis besides apoptosis, necroptosis, and pyroptosis [24, 25]. Here, we find that PARP-1 inhibitors (3-AB) significantly inhibited LPS-induced cell death, suggesting the presence of parthanatos in endotoxemia. In addition, both 3-AB and erlotinib effectively inhibited PAPR-1 activation, PAR accumulation, and translocation of AIF into the nucleus in LPS-treated RAW264.7 cells. Meanwhile, LPS lost its ability to activate PARP-1 in *TLR4*^*–/–*^ BMDM. This study presented new evidence that erlotinib mediated LPS-induced parthanatos via inhibited TLR4 translocation. All these results indicated that cell surface TLR4 expression is essential for LPS-induced parthanatos in macrophage (Fig. 6).

Parthanatos is a form of programmed cell death induced by the overactivation of PARP-1 due to extensive DNA damage, and finally promotes large-scale DNA fragmentation and chromatin condensation [1, 26, 27]. Accumulating evidence suggest that PARP-1-dependent cell death (parthanatos) is closely related to the excess generation of ROS [6, 7]. A large number of studies proven that ROS have been well set up in parthanatos [6, 28–30]. LPS have been otherwise reported to be capable of inducing PARP-1 activation [31–33]. Some studies have demonstrated that in LPS treatment, monocytes and macrophages can produce large amount of ROS that in turn activates MAPK signaling cascades and nuclear factor NF-κB [34–37]. There is a close relationship between inflammatory response and oxidative stress.

TLR4 expression on cell surface of innate immune cells plays an indispensable role in regulating the progression of host reaction and inflammation in bacterial infection by activating signal transduction pathways [38, 39]. After activation, TLR4 leads to synthesis of reactive oxygen and nitrogen species and oxidative and nitrosation stress and therefore diseases related to TLR mechanism [40]. In our study, the production of ROS is increased in response to LPS, and erlotinib pretreatment could reverse the result. However, there is no direct evidence whether LPS-induced ROS production can directly activate PARP-1. We also found that erlotinib could inhibit the cells surface expression of TLR4 and activation of downstream MAPK and PI3K signal pathway after LPS treatment. Perhaps it explains why erlotinib can suppress the activation of PARP-1. However, we do not demonstrate a direct association between late period cell surface TLR4 increase and PAPR-1 activation, and that we are more likely to believe that the PI3K/Akt pathway is involved in the late-phase TLR4-mediated inflammatory response, but the exact mechanism is still unknown.

EGFR belongs to the ERBB family and is a growth factor receptor [41, 42]. Recently, more and more studies focus on the crosstalk between LPS-TLR4 and EGFR signal pathway. Basu et al. reported that the *Helicobacter pylori* secretory protein HP0175 binds to TLR4 and transactivates EGFR in human gastric epithelial cells [43]. Bromberg et al. reported that TLR4 binds to EGFR in response to acute O3-induced neutrophilic airways inflammation [44]. And our previous study demonstrated that the change in TLR4 surface expression is mainly due to EGFR-mediated translocation, and LPS-induced EGFR phosphorylation and subsequent concurrent internalization of EGFR and TLR4 were essential for receptor endocytosis [18]. Even so, there is no evidence of a direct relationship between TLR4 and EGFR. Here, we found that the EGFR inhibitor erlotinib could inhibit parthanatos, and EGFR may also be participated in the regulation of LPS-induced parthanatos. Meanwhile, the exact mechanism of the interaction between EGFR and TLR4, and whether they co-regulate parthanatos, therefore remains to be further explored.

In conclusion, we have found that the occurrence of parthanatos in endotoxemia and erlotinib can significantly mitigate LPS-induced parthanatos in vivo and in vitro via suppressing cell surface TLR4 expression. All these results indicate that TLR4 plays an significant role in parthanatos in the presence of LPS, and PARP-1 probably represents a latent new target for the treatment of endotoxemia.

## Materials and methods

### Animals

C57BL/6 (WT) mice were purchased from Guangzhou Dean Gene Technology Co., Ltd (Guangzhou, China), TLR4 knockout mice were purchased from GemPharmatech (Jiangsu, China). Mice aged 8 weeks with an average body weight of 25 g were used. For animal studies, the mice were divided into the following four groups randomly: (1) negative control group: mice were intraperitoneally injected with normal saline or pretreated with equal amounts of erlotinib solvent (Captisol) orally 2 h before saline i.p.; (2) LPS group: mice received LPS (20 mg kg^−1^, i.p.) treatment alone; (3) LPS + erlotinib group: mice were administered intragastrically with 100 mg/kg erlotinib 2 h before LPS (20 mg kg^−1^, i.p.) injection; and (4) LPS + 3-AB group: mice were pretreated with 3-AB (30 mg kg^−1^, i.p.) 1 h before LPS (20 mg kg^−1^, i.p.) injection. All animal experiments were reviewed and approved by the Animal Ethics Committee of Guangdong Medical University. 3M’s animal research programs follow the Guidelines for the Care and Use of Laboratory Animals and the Animal Welfare Act. The investigator was blinded to the group allocation when assessing the survival rate outcome.

### Culture of bone marrow-derived macrophage (BMDM) and cells

BMDM were harvested from both the femurs and tibias of 8-week-old male mice, as previously described [45]. The macrophage cell lines RAW264.7 cells (C7505) were purchased from the Beyotime Biotechnology (Shanghai, China). Cells were cultured with DMEM containing 5% Australian fetal bovine serum.

### Flow cytometry

Peritoneal macrophages were stained with macrophage surface marker F4/80 for 30 min. For measuring the cell surface TLR4 expression, single-cell suspensions were stained with CD284 (TLR4) antibody (12-9041-80, eBioscience, San Diego, CA, USA) at 4 °C for 30 min. In order to detect cell death, cells were resuspended in binding buffer and incubated with FITC Annexin (BD Pharmingen, San Jose, CA, USA) for 15 min and then added with 7AAD for 5 min in dark. The double-stained cells indicated the death of cells. Cells were evaluated using FACScanto cytometer (BD Biosciences, San Jose, CA, USA) and 20,000 events were collected. All samples were analyzed using FlowJo V10 software (TreeStar, Ashland, OR, USA).

### Reactive oxygen species measurement

RAW264.7 cells or BMDM seeded at 2 × 10^5^ cells/well in 6-cm petri dish were cultured and pretreated with indicated reagents. At the end of LPS (1 μg/ml) treatment, cells were loaded with fluorescent dye DCFH2-DA (Beyotime Biotechnology, China) in serum-free DMEM and incubated for 30 min at 37 °C in the dark. Then, cells were washed twice with PBS after suspended in fresh DMEM; the fluorescence intensity was measured by FACScanto cytometer. Data were analyzed using FlowJo V10 (TreeStar).

### RNA analysis by quantitative real-time PCR

Total RNA was isolated from cells using TRIzol RNA Isolation Reagents (Takara, China). PrimeScript^TM^ RT reagent Kit with gDNA Eraser (RR047A, TaKaRa, China) was used for performing cDNA synthesis. mRNA was quantified with using TB Green Premix Ex Taq^TM^ II (RR820A, TaKaRa, China). All experimental doses were in accordance with the manufacturer’s protocols. StepOne^TM^ system (Thermo Fisher Scientific, Pittsburgh, PA, USA) was used for performing real-time PCR. The list of primers was as followed: TNF-α forward: 5’-AAGCCTGTAGCCCACGTCGTA-3’, reverse: 5’-GGCACCACTAGTTGGTTGTCTTTG-3’. IL-1β forward: 5’-GCAACTGTTCCTGAACTCAACT-3’, reverse: 5’-ATCTTTTGGGGTCCGTCAACT-3’. The final data were analyzed using Ct method and calculated and expressed using an equation (2^–ΔΔCt^), which provides the amount of the target that was normalized into an internal reference. Ct value represents the number of cycles required for fluorescence intensity to reach the threshold value in PCR process.

### Immunofluorescence

Cells grown on the immunofluorescence confocal dish were fixed using 4% paraformaldehyde for 15 min, and permeabilized with 0.5% Triton-X-100 for 10 min, followed by blocked with 5% BSA for 30 min under the room temp. Then, the anti-AIF antibody was added and incubated overnight at 4 °C. Appropriate fluorescent secondary antibody was used for 1 h at room temp in the dark. Nuclei were counterstained with DAPI and then sealed with anti-fluorescence quenching agent before images were collected under the laser fluorescence microscopy (Olympus FV1000, Japan).

### Antibodies and reagents

Antibodies: PARP-1 (9532), AIF (5318), GAPDH (2118), Lamin B (12586), p-AKT (4060), p-P38 (4511), p-ERK1/2 (4370), AKT (4691), P38 (8690), and ERK1/2 (4695) were obtained from Cell Signaling Technology (Danvers, MA, USA). Goat anti-rabbit IgG (H&L) (HS101-01) were obtained from TransGen Biotech (Beijing, China). PAR antibody (AM80) was from Merck Millipore (CA, USA). Alexa Fluor 488 goat anti-rabbit (A-11034) secondary antibodies were obtained from Thermo Fisher Scientific (Pittsburgh, PA, USA). GAPDH (60004-1-Ig) was purchased from Proteintech (Wuhan, Hubei, China).

Reagents: LPS (L4391) was purchased from Sigma (Louis, MO, USA), erlotinib (S1023) and 3-AB (S1132) were obtained from Selleck.cn (Shanghai, China).

### Statistical analysis

All data were presented as mean ± SD and performed at least three times. Differences between two groups were analyzed using Student’s *t* test. One-way ANOVA was used for comparing multiple groups. *χ*^2^ test was used for survival studies. *P* values of <0.05 were considered statistically significant. All statistical analyses and graphs were completed with Prism GraphPad 6.0 (GraphPad software, CA, USA).
